# Reactivation of HIC-1 gene by saRNA inhibits clonogenicity and invasiveness in breast cancer cells

**DOI:** 10.3892/ol.2014.2633

**Published:** 2014-10-24

**Authors:** FENG ZHAO, SHENGLI PAN, YAN GU, SHANYU GUO, QIANCHENG DAI, YINGYAN YU, WEI ZHANG

**Affiliations:** 1 Department of Surgery, The Ninth People’s Hospital of Shanghai Jiao Tong University, School of Medicine, Shanghai 200011, P.R. China; 2 Department of Surgery, Shanghai Ruijin Hospital of Shanghai Jiao Tong University, School of Medicine, Shanghai 200025, P.R. China

**Keywords:** breast cancer, HIC-1, clonogenicity, cell invasion, saRNA

## Abstract

Hypermethylated in cancer 1 (HIC-1) is a tumor suppressor gene, which is epigenetically silenced in breast cancer. It is known that the loss of HIC-1, caused by promoter hypermethylation, is associated with tumor aggression and poor survival in breast carcinoma. It has been shown that small activating RNA (saRNA) targeting promoter sequences may induce gene re-expression. In the current study, saRNA was used to restore HIC-1 expression, and the effects on colony formation, invasiveness and the cell cycle in breast cancer cells were explored. dsHIC1-2998, an saRNA, exhibited activating efficacy on MCF-7 and MDA-MB-231 cancer cell lines. A clonogenicity assay showed that evident colony inhibition was induced via saRNA-mediated re-expression of HIC-1 in the two cancer cell lines. Reactivation of HIC-1 significantly inhibited cell migration and invasion, resulting in G0/G1 cell cycle arrest in these cell lines. These findings suggest that HIC-1 may be a potential target in gene therapy for the treatment of breast cancer. saRNA may function as a therapeutic option for upregulating tumor suppressor genes in breast cancer.

## Introduction

Breast cancer is one of the most common malignancies worldwide. It is desirable to explore new molecular targets and develop novel targeted drugs for breast cancer patients. Hypermethylated in cancer 1 (HIC-1), a tumor suppressor gene for breast cancer, located on 17p13.3, encodes a transcriptional suppressor protein, with five Kruppel-like C_2_H_2_ zinc finger motifs and the N-terminal protein-protein interaction domain, BTB/POZ (1). Epigenetic silencing of HIC-1 is significant in the pathogenesis of epithelial cancers. The loss of HIC-1 may be closely associated with the promotion of tumorigenesis in a wide variety of cell types. Decreased expression of the HIC-1 gene is observed in non-small cell lung cancer (2), hepatocellular carcinoma (3), gastric cancer (4) and human medulloblastomas (5), and the loss of HIC-1 expression is a common event in primary breast cancer (6). Inactivation of HIC-1 in breast carcinomas is associated with tumor metastasis (7), and a previous study demonstrated that restoring the HIC-1 expression by demethylation treatment impaired the aggressiveness of head and neck squamous cell carcinoma (8).

In 2006, Li *et al* (9) reported a novel function of double-stranded RNA (dsRNA) molecules. The results indicated that dsRNA induced sequence-specific transcriptional activation by targeting gene promoter regions. This phenomenon was termed RNA-induced gene activation (RNAa) and the dsRNA molecules were denominated as small activating RNAs (saRNAs). Janowski *et al* (10) reported similar findings, in which multiple duplex RNAs, complementary to the progesterone receptor (PR) promoter, activated PR protein expression in T47D and MCF-7 human breast cancer cells (10). Subsequently, dsRNAs have been used for the activation of various target genes in multiple laboratories. Chen *et al* (11) and Yang *et al* (12) used dsRNAs to upregulate p21WAF1/CIP1 (p21) in human bladder cancer cells. In addition, Ren *et al* (13) induced NKX3-1 in prostate tumor cells by saRNA. More recently, our research group successfully reactivated the HIC-1 tumor suppressor in gastric cancer and in breast cancer (4,14). Our previous studies disclosed that dsHIC1-2998, an saRNA, effectively activated HIC-1 with evident suppression of cell growth and induction of apoptosis in breast cancer. These findings indicate the possibility that this saRNA may become a practical and highly cost-effective approach for gene therapy.

In the present study, the efficacy of saRNA on suppression of clonogenicity and invasiveness of breast cancer cell lines was investigated by the reactivation of HIC-1. dsHIC1-2998, which targets the HIC-1 promoter region, was used as an effective saRNA. This study aims to increase the supporting evidence for saRNA as a promising molecule for restoring the gene expression and biological activity of tumor suppressors in breast cancer.

## Materials and methods

### saRNA design

The dsRNA targeting the region (2998 bp) above from the transcription start site of human HIC-1 was designed based on the rational design rules described (9,15) and our previous reports (4,14). The sequences of saRNA-HIC-1 (dsHIC-1-2998) used in this experiment were as follows: Sense, 5′-CGGUUUCCMGGAGAAGUUATT-3′ and antisense, 5′-UAACUUCUCCAGGAAACCGTT-3′. A further RNA strand, unrelated to that of the human dsRNA sequence, was used as a control (sense, 5′-ACGMGACACGUUCGGAGAATT-3′ and antisense, 5′-UUCUCCGAACGMGUCACGUTT-3′). All dsRNA sequences were synthesized by Genepharma Inc. (Shanghai, China).

### Cell culture and saRNA transfection

MCF-7 and MDA-MB-231 breast cancer cell lines were originally obtained from the Institute of Biochemistry and Cell Biology, Shanghai Chinese Academy of Science (Shanghai, China). MCF-7 and MDA-MB-231 cells were cultured in Dulbecco’s modified Eagle’s medium with 10% fetal bovine serum (Gibco-BRL, Invitrogen Life Technologies, Carlsbad, CA, USA). Immediately prior to transfection, the cells were trypsinized, diluted with growth medium without antibiotics or serum, and seeded into six-well plates at a density of 3.0×10^5^ cells per well for MCF-7 cells and 4.0×10^5^ cells per well for MDA-MB-231 cells. The transfection of saRNA and control RNA was conducted at a concentration of 50 nmol/l using Lipofectamine 2000 (Invitrogen Life Technologies) according to the manufacturer’s reverse transfection instructions. The cells were harvested for further analysis. In this study, the mock group was transfected with lipofectamine 2000 alone, while the control group was transfected with non-specific dsRNA.

### mRNA analysis by real-time polymerase chain reaction (PCR)

Total RNA was extracted using TRIzol solution (Invitrogen Life Technologies). Reverse transcription PCR was performed in a 20-μl reaction system according to the manufacturer’s instructions (Promega Corporation, Madison, WI, USA). The cDNA was amplified using gene-specific primer sets in conjunction with the SYBR Green PCR master mix (Applied Biosystems, Foster City, CA, USA). Real-time PCR was performed in a reaction mixture with a final volume of 20 μl containing 10 μl SYBR Green PCR Master Mix, 1 μl of 5 mmol/l paired primer specific to the target gene and 1 μl cDNA. The primers used for real-time PCR were as follows: Forward, 5′-GACGGCGACGACTACAAGAG-3′ and reverse, 5′-GAATGCACACGTACAGGTTGTC-3′ for HIC-1; and forward, 5′-GGACCTGACCTGCCGTCTAG-3′ and reverse, 5′-GTAGCCCAGGATGCCCTTGA-3′ for GAPDH.

### Protein analysis by western blotting

The cells were harvested and washed twice with PBS, pH 7.4, and resuspended in lysis buffer [1 mM dithiothreitol, 0.125 mM EDTA, 5% glycerol, 1 mM phenylmethyl 5 μl fonylfluoride, 1 μg/ml leupeptin, 1 μg/ml pepstatin, 1 μg/ml aprotinin, 1% Triton X-100 (Shanghai Chemical Co., Shanghai, China) in 12.5 mM Tris-HCl buffer, pH 7.0] on ice. The cell extracts were centrifuged and the protein concentration was determined using the bicinchoninic acid protein assay kit (Pierce Biotechnology, Inc., Rockford, IL, USA) according to the manufacturer’s instructions. Each protein extract (50 μg) was electrophoresed on a 12.5% SDS-polyacrylamide gel, transferred to polyvinylidene difluoride membranes in a buffer containing 25 mM Tris-HCl, pH 8.3, 192 mM glycine and 20% (v/v) methanol, and blocked in 5% (w/v) skimmed milk in Tris-buffered saline-Tween 20 (TBST; 0.1% v/v) for 2 h at room temperature. This was subsequently probed with specific primary antibodies (mouse monoclonal anti-HIC-1, 1:800, ab55120, Abcam, Cambridge, England; and mouse monoclonal anti-GAPDH, 1:5000; GW22763, Sigma-Aldrich, St. Louis, MO, USA) overnight at 4°C. The primary antibodies were removed and the blots were extensively washed with TBST three times. The blots were incubated for 1 h at room temperature with horseradish peroxidase-conjugated rabbit anit-mouse polyclonal secondary antibody (1:5000; Sigma-Aldrich) in TBST. Following this, the blots were washed for 30 min and developed using an Enhanced Chemiluminescence kit (NENTM Life Science Products Inc., Boston, MA, USA).

### Clonogenicity assay

The cancer cells were transfected with saRNA or control RNA for 12 h, and then transferred to six-well plates and seeded at a density of 1.0×10^3^ cells per well. The plates were incubated at 37°C in a humidified atmosphere of 5% CO_2_ for 12 days. The culture medium was changed every three days. Clonogenicity was analyzed at 12 days following the saRNA transfection. The plates were stained with 0.05% crystal violet solution for 15 min, and the colonies were counted under the inverted microscope and photographed. The experiments were performed in triplicate, at minimum. Data are presented as the mean ± standard deviation (SD).

### Scratch-healing assay

MDA-MB-231 and MCF-7 cells were seeded into six-well plates at a density of 0.8×10^5^ cells per well. Following overnight incubation, the cells were transfected with 50 nmol/l saRNA-HIC-1 or control RNA for 72 h until the cells reached full confluence. A monolayer of cells was scratched by a 1-mm micropipette tip, rinsed with PBS to remove cell debris and cultured continuously in growth medium containing 1% fetal bovine serum (Gibco-BRL, Invitrogen Life Technologies). The wound-closing procedure was observed for 72 h. The wound width of each well was calculated at every 24 h interval.

### Invasion and migration assays

The cells were harvested following the 72-h transfection of saRNA-HIC-1 or the control RNA, and were resuspended in medium. For the MCF-7 cells, the cell concentrations for the migration and invasion assay were 1.0×10^6^ and 2.5×10^6^ cells/ml, respectively. For the MDA-MB-231 cells, the cell concentrations for the migration and invasion assays were 1.5×10^5^ and 5×10^5^ cells/ml, respectively. In total, 0.2 ml cells was added to the top Transwell chamber (24-well insert, 8-μm pore size; Millipore, Bedford, MA, USA) and 0.6 ml medium with 10% fetal bovine serum was added to the lower chamber as a chemoattractive factor. Subsequently, the cells were incubated for 20–48 h. The cells that did not migrate through the pores were removed by scraping the upper surface of the membrane with a cotton swab. The cells that migrated to the lower surface of the membrane were fixed with 100% methanol for 15 min and stained with 0.1% crystal violet for a further 15 min. The cells that migrated through the insert were counted at five random fields and expressed as the mean number of cells per field. These experiments were performed in triplicate.

### Cell cycle analysis by flow cytometry

The cells (1×10^6^ cells/ml) were transfected with saRNA or control RNA. At 96 h following transfection, the cells were harvested and fixed in 70% ethanol at −20°C overnight, and then stained with 250 μg/ml propidium iodide (Sigma-Aldrich), 5 μg/ml RNase A (Sigma-Aldrich) and 5 mmol/l EDTA in PBS (pH 7.4) for 30 min. The cell cycle analysis was performed using the FACScan (Beckman Instruments, Fullerton, CA, USA). The data was evaluated using the FlowJo software (Tree Star, Inc. Ashland, OR, USA).

### Statistical analysis

The results are presented as the mean ± SD. Statistical analyses were performed using SPSS, version 15.0 (SPSS Inc., Chicago, IL, USA). Student’s t-test and one-way analysis of variance, followed by Dunnett’s multiple comparison tests, were conducted. P<0.05 was considered to indicate a statistically significant difference, indicated by asterisks in the figures.

## Results

### Reactivation of HIC-1 inhibits colony formation of breast cancer cells

**Figure 1:**
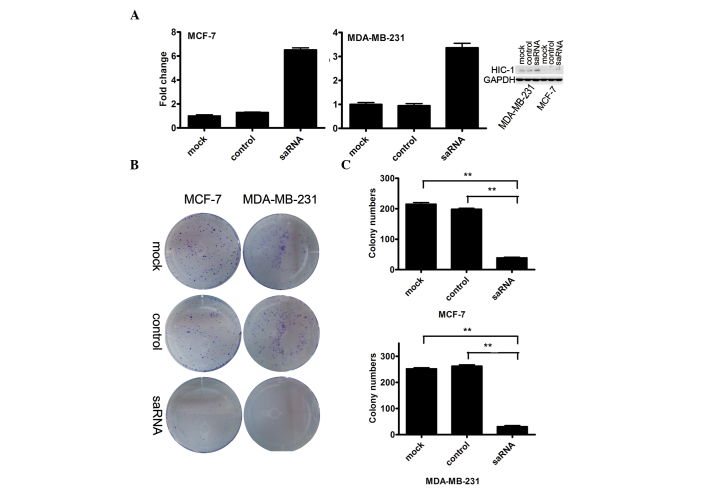
Upregulation of HIC-1 by saRNA-HIC-1 inhibits colony formation in breast cancer cells. (A) saRNA-HIC-1 effectively restored HIC-1 mRNA and protein expression in MCF-7 and MDA-MB-231 cells. (B) The number and size of colonies in saRNA-HIC-1 group were smaller than those in the control groups. (C) The bar charts demonstrate that the number of colonies in the saRNA-HIC-1 group was significantly fewer than in the control RNA group (P<0.05). The columns represent the mean ± the standard deviation of triplicates. ^**^P<0.01 vs. the mock or control group, respectively. HIC-1, hypermethylated in cancer 1; saRNA, small activating RNA.

Initially, whether or not dsHIC1-2998 was an effective saRNA was investigated. In total, 50 nmol/l saRNA was transfected into MDA-MB-231 and MCF-7 cancer cell lines. The restoration of HIC-1 mRNA was evaluated by real-time RT-PCR 96 h following saRNA transfection. In MCF-7 cells transfected with HIC-1 mRNA, the HIC-1 mRNA level was upregulated 6.52-fold compared with the mock-transfected cells. In MDA-MB-231 cells transfected with HIC-1 mRNA, the HIC-1 mRNA level was upregulated 3.37-fold, compared with the mock-transfected cells. The protein analysis revealed that HIC-1 protein levels were also elevated based on the saRNA transfection for the two cancer cell lines (Fig. 1A), compared with that of the control cells. Therefore, dsHIC1-2998 was confirmed as effective saRNA-HIC-1.

Subsequently, 50 nmol/l saRNA-HIC-1 or control RNA was transfected into MCF-7 and MDA-MB-231 cells for 12 h and the clonogenicity was analyzed at 12 days following the saRNA transfection. The size of the colonies formed in saRNA-HIC-1 group was smaller than that in the control groups (Fig. 1B). The colonies containing at least 50 cells in five fields were randomly counted. The number of colonies was significantly reduced in the saRNA-HIC-1 transfection group in MCF-7 cells (39.0 vs. 198.7 and 215.2; the mock and control groups, respectively; P<0.001) and MDA-MB-231 cells (31.0 vs. 262.7 and 252.3; the mock and control groups, respectively, P<0.001), compared with the control groups (Fig. 1C).

### Reactivation of HIC-1 inhibits cell migration and cell invasion of breast cancer cells

**Figure 2:**
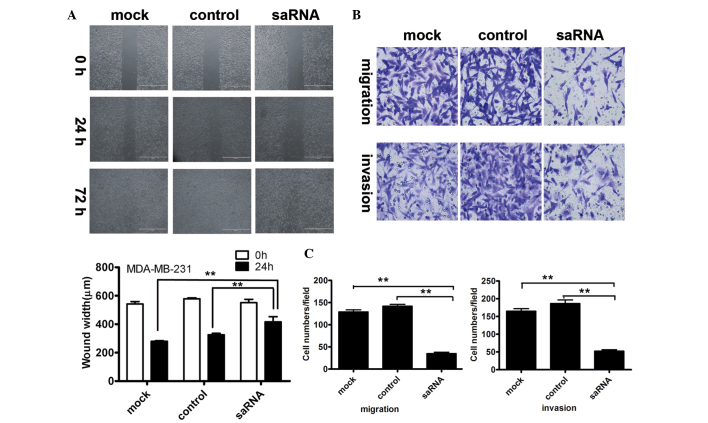
Upregulation of HIC-1 suppresses cell migration and invasion in the MDA-MB-231 cell line. (A) Wound closure assay revealed a clear inhibiting effect on cell migration by serial observation at 24 and 72 h of the saRNA-HIC-1 group (magnification, ×40). The wound widths of MDA-MB-231 migration at 24 h were compared between the saRNA-HIC-1 group, the non-specific control and mock-transfected cells (281.43 vs.453.91 and 461.44 μm; P<0.05). (B) Migration: Transwell chamber assay for cell migration. Following the transfection of saRNA-HIC-1 for 72 h, the tumor cells that migrated to the lower chamber were calculated after 20 h induction. Invasion: Following the transfection of saRNA-HIC-1 for 72 h, the tumor cells that passed through Transwell membrane to the lower chamber were calculated after 20 h induction. The cells were stained and counted under light microscopy and photographed at magnification, ×100. (C) The bar charts represent the mean ± the standard deviation from three independent experiments for different groups of the cell migration assay (left) and cell invasion assay (right). ^**^P<0.01 vs. the mock or control group, respectively. HIC-1, hypermethylated in cancer 1; saRNA, small activating RNA.

Initially, the cell migration ability was analyzed using a wound closure experiment in MDA-MB-231 cells and MCF-7 cells following saRNA-HIC-1 transfection. The wound-closing procedure was serially observed for 72 h following the introduction of the wound on the plate. As shown in Fig. 2A, the speed of wound-closing was slower in saRNA-HIC-1-transfected cells, compared with that in the control groups (untransfected and mock-transfected HIC-1 cells). At 72 h, the wounds of the control groups were completely closed. This indicated that the upregulation of HIC-1 expression inhibited cell migration *in vitro*. The results for the MCF-7 cell line could not be obtained due to its low migration capacity.

Following this, the cell migration and invasion ability were analyzed for the saRNA-HIC-1 transfectant on MDA-MB-231 and MCF-7 cell lines using a Transwell chamber. As shown in Fig. 2B, MDA-MB-231 cells in the saRNA-HIC-1 group exhibited a weaker migration ability with fewer cells compared with the control groups. The cells in the saRNA-HIC-1 group exhibited weaker invasive ability, with fewer cells compared with the control groups. The cell counting revealed that cell numbers for cell migration (35 vs. 142 and 129; the mock and control groups, respectively; P<0.001) or cell invasion (52.3 vs*.* 186.7 and 165; the mock and control groups, respectively; P<0.001) in saRNA-HIC-1 group were significantly lower than that of the control groups (Fig. 2C). The results for the MCF-7 cell line could not be obtained due to its low migration and invasion capacity.

### Upregulation of HIC-1 expression via saRNA induces cell cycle arrest in breast cancer cells

**Figure 3:**
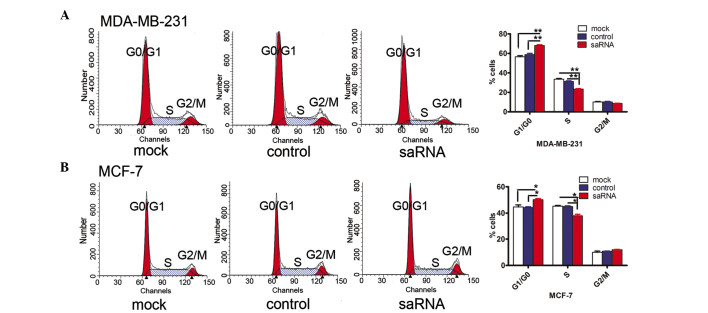
Effect of saRNA-HIC-1 transfection on the cell cycle. saRNA-HIC-1-transfected (A) MDA-MB-231 and (B) MCF-7 cells showed an increased percentage of G0/G1 phase cells and a decreased percentage of S phase cells. ^*^P<0.05 and ^**^P<0.01 vs. mock or control group, respectively. HIC-1, hypermethylated in cancer 1; saRNA, small activating RNA.

The cell cycle fraction was investigated using flow cytometry based on saRNA-HIC-1 transfection for 96 h for the two cancer cell lines. In the MDA-MB-231 cells, saRNA-HIC-1 transfection caused a significant increase in the G1/G0 fraction (68.64 vs. 59.64 and 55.63%; the mock and control groups, respectively) with concurrent decline in S (22.89 vs. 30.58 and 33.94%) and G2/M fractions (8.47 vs. 9.78 and 10.43%; the mock and control groups, respectively), compared with the controls (Fig. 3). However, a significant increase in the G1/G0 fraction (50.79 vs. 43.75 and 43.63%; the mock and control groups, respectively) with a concurrent decline in the S fraction (37.07 vs. 45.38 and 44.90%; the mock and control groups, respectively) and slight increase in the G2/M fraction (12.14 vs. 10.87 and 9.27%; the mock and control groups, respectively) were observed in the MCF-7 cells. Overall, these results indicated that the reactivation of the HIC-1 gene by saRNA induces G1/G0 phase arrest.

## Discussion

It is known that short 21-nucleotide dsRNA molecules may silence endogenous human genes in a sequence-specific manner. This method, termed RNA interference (RNAi), develops rapidly and is extensively used in experimental medicine. RNAi exhibits significant capacity in the silencing of oncogenes. The mechanism behind RNAi involves the knockdown of endogenous human genes. RNAi has exhibited potential in the field of tumor therapy; however, no dependable method has been established for the restoration of endogenous tumor suppressor genes, with the exception of vector-mediated gene engineering. RNAa is mediated by small dsRNA fragments. The RNAa technology exhibits the opposite efficacy of RNAi by activating, as opposed to silencing, the target genes. As a novel technique, RNAa has successfully activated several target genes in various human diseases including those involved in cancers, such as p21, E-cadherin, VEGF, WT1 and several others (16–21). Although the exact mechanism of RNAa remains unclear, as a new molecular tool, saRNA is currently under use in the study of gene function and has exhibited promising initial results. Mao *et al* (22) reported that the upregulation of E-cadherin by saRNA inhibits cell invasion and migration of 5637 human bladder cancer cells. Restoring the E-cadherin gene in MDA-MB-453 breast cancer cells induced apoptosis and inhibited cell proliferation (21). Activation of the p21 gene in a variety of cancer cells, including prostate, bladder, liver, pancreas and lung cancer cells, inhibited cell proliferation and clonogenicity (18,19,23,24). Restoration of the p21 gene enhanced apoptotic cell death and caused G0/G1 arrest in T24 and J82 bladder cancer cells (11). Recently, lipid nanoparticle-formulated dsp21-322-2′F revealed an inhibiting effect on bladder tumors *in vivo* (25). saRNAs have exhibited similar benefits to RNAi as a therapeutic molecule.

HIC-1 is a transcriptional repressor involved in the regulation of growth control, cell survival and DNA damage response (26). HIC-1 has been observed to be epigenetically silenced in human cancers including breast cancer (27). Hypermethylation is a significant inactivation mechanism for a number of tumor suppressors. Boulay *et al* revealed that the loss of HIC-1 is involved in stress-induced migration and invasion in breast cancer (7). HIC-1 promoter hypermethylation is associated with tumor aggressiveness and poor survival. The restoration of HIC-1 expression by a demethylation reagent, caused the suppression of cancer progression in head and neck squamous cell carcinoma (8).

Our research group has had an interest in this novel molecular technique since it was established. As demonstrated in our previous studies, the HIC-1 tumor suppressor was initially reactivated in gastric cancer cells. The reactivation of HIC-1 was observed to suppress cell migration and induce cell cycle arrest in the G0/G1 phase as well as apoptosis (4). Subsequently, the HIC-1 tumor suppressor was successfully reactivated in breast cancer cells. dsHIC1-2998 was further confirmed as effective saRNA for gastric cancer and breast cancer cells. The saRNA-HIC-1 effectively activated the HIC-1 gene with evident suppression of cell growth and induction of apoptosis in breast cancer (14). In the current study, further evidence has been obtained, confirming that saRNA-HIC-1 effectively inhibits clonogenicity in both MCF-7 and MDA-MB-231 cells. However, the change of invasiveness in MDA-MB-231 cells is based on HIC-1 activation, while the change of invasiveness of MCF-7 cells is unclear. The result reflects how different cell lines have varying biological behavious.

In conclusion, cell models were created for the restoration of the tumor suppressor gene, HIC-1, in breast cancer cells. Using these cell models, the effects of upregulating the HIC-1 gene were explored in multiple biological features, including tumor growth, migration, invasion and the cell cycle. These findings provide evidence that HIC-1 may potentially be a target for gene therapy against breast cancer. The upregulation of HIC-1 by saRNA molecules may be a therapeutic strategy for the suppression of breast cancer progression. The targeted activation of tumor suppressor genes by saRNA may provide a new therapeutic option that could significantly improve the treatment of breast cancer.
